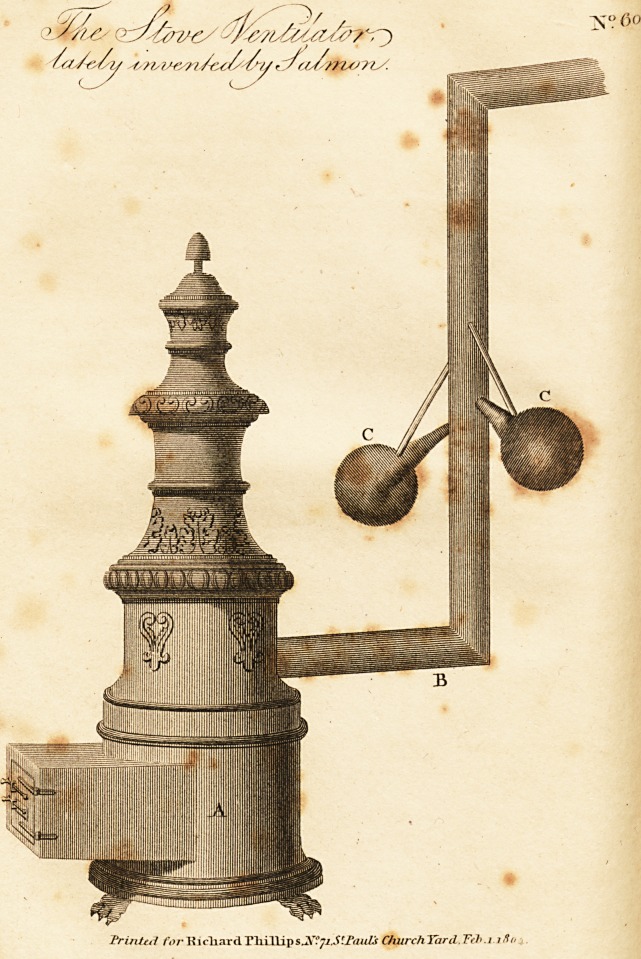# Instructions on the Means of Maintaining the Salubrity and Purifying the Air of the Wards of Hospitals and Crowded Assemblies

**Published:** 1804-02-01

**Authors:** 

**Affiliations:** Secretary


### Medical and Physical Journal.

VOL. XI.] FEBRUARY 1, 1804. [NO. LX.

*Printed for R. PHILLIPS, by W. Thorne, Red Lion Court, Fleet Street, London.*

### *To the Editors of the Medical and Physical Journal.*

FAS EST AB HOSTE DOCERI.

GENTLEMEN,

THE enclosed Instructions on the means of maintaining the salubrity and purifying the air of the wards of hospitals, applicable also to large assemblages of people in courts of justice, playhouses, &c. drawn up by the French Council of Health in the year 1795, appear to me to hold out a promise of infinite utility at the present moment, when a regard to our preservation and defence has assembled such powerful fleets and armies, the valuable members of which are exposed, among other perils, to the calamities it is the object of these instructions to avert. You will find them to contain several important observations on the employment of the gases, in destroying contagion, a subject which has been rendered more than usually interesting by a late award of the British parliament.

I am, &c.

*Pentonville, Dec. 19, 1803.* S. J.

### INSTRUCTIONS ON THE MEANS OF MAINTAINING THE SALUBRITY AND PURIFYING THE AIR OF THE WARDS OF HOSPITALS AND CROWDED ASSEMBLIES.

[With an Engraving.]

### *Means of Cleanliness.*

CLEANLINESS, which is so essential under all the circumstances of life, is the most powerful corrective of the local defects of salubrity, and ought therefore to constitute the principal object of the attention of all hospital agents. Professional men, whose duty it more particularly is to enforce a strict observance of every necessary precaution, should endeavour to convince the sick of the direct rect influence which cleanliness has on the complete and speedy re-establishment of their health. Persuasion has a powerful effect on men, when it is founded on the opinion of the interest that is taken in their health and preservation. The regulations which follow are, for the greater part, applicable not merely to hospitals but to prisons, and, in general, to all establishments where many individuals, some in a healthy, and others in a diseased state, may be crowded together.

As soon as the sick are brought into the hospital, their hands and feet should be washed in warm water.

The utensils destined for their different uses ought to be frequently washed.

The foul linen should be carried to the most airy part of the building, and there suspended on solid poles, without being heaped together, until the time arrives when it is to be washed. The pieces of linen which may have been employed in the dressing of wounds, &c. ought to be instantly collected in baskets, and steeped in water till they can be washed out in a strong lie.

The great coats and blankets should be beaten and brushed from time to time, and afterwards fumigated with burning sulphur. They ought to be sent to the oven at least once a year.

The wool belonging to the mattrasses should be beaten and carded, as far as it is practicable, every six months. Their coverings, and those belonging to the straw mattrasses, ought to be frequently and effectually washed in lie.

The bathing tubs, if wood be the material employed, should be painted and varnished both within and without.

In each of the wards vessels filled with water should be placed, and their contents frequently renewed.

The vinegar, which it has been the practice to bestow uselessly on fumigations, should be mixed with water, and employed in gargles, or in sprinkling the floors of the wards, before they are swept.

The walls and cielings of the wards ought to be white-washed at least once a year; and the bedsteads, window frames, tables, and even planks, washed with lime-water, or with a strong alkaline lie.

The articles of clothing, &c. belonging to the hospital, which may have been worn by a patient labouring under a contagious disease, should not be again employed, until after they have been sweetened by the means which will be pointed out in the sequel.

Each

Each of the lamps ought to be provided with a conductor, to facilitate the escape of the smoke withoutside.

The number of the beds contained in each ward ought to be irrevocably fixed, and inscribed over the door. It ought to be governed by the extent, form, elevation, and disposition of the ward, in such a way as that, in a ward the cieling of which may have an elevation of from ten to twelve feet, the beds should be stationed at least two feet from each other. If it should have an elevation of nine feet only, the space ought to be augmented to two feet and a half. Between each of the beds and the wall, a space of from two to three feet ought to be allowed. Whatever the extent of the ward may be, ranges of beds ought in no case to be allowed in the centre.

The patients should not be suffered to pass reciprocally to and from the wards in which contagious diseases are prevalent.

In the vicinity of the wards, or of the hospital, neither stagnant water, heaps of dung, nor any vegetable or animal substances in a decomposed state ought to be allowed.

Close-stools should be constantly provided in such numbers as that those in use may be instantly replaced whenever necessity may require. They ought not, however, to be allowed unless to the patients who are dangerously ill. Water should be constantly kept in these utensils, the seat of which ought to be carefully washed from time to time. They should be covered, both without and within, with a thick layer of drying oil.

The improper position of the necessaries, in the greater part of the hospitals, is one of the most direct causes of the disagreeable smell which strikes the organs on entering. Notwithstanding an attempt has been made, in several places, to cleanse them and drain off their contents, by the means of a current water, still it frequently happens that the water has not a sufficent impulsive force for this purpose, while in other cases it is not to be procured. Another very common defect consists in the doors of the necessaries not being provided with weights withinside, that may enable them to shut of themselves. It very seldom occurs that the expedient has been resorted to of forming, between them and the wards, an intermediate vestibule with transverse and corresponding windows, calculated to renew the air constantly, and to intercept the communication of the smell. These precautions are, however, best calculated to diminish the influence of the infection which is exhaled from the vicinity of the necessaries. It would perhaps be

 possible possible to remedy this inconvenience by removing the pits, or cavities, to a distance of at least six feet from the walls; and by constructing, on each floor of the building, a privy to which the patients may repair by a slight but sufficiently solid gallery. In each of these privies there should be five or six seats placed circularly over the pits.

The seats of the necessaries ought to be washed daily; and this article of cleanliness should be made a very strict regulation of police.

*Mechanical Means.*

The best mode of preventing, or correcting, the bad qualities contracted by the air in the wards of hospitals, is to introduce it from without, at the same time that an outlet is afforded to that which has been vitiated by the respiration, and by the emanations from the sick, more particularly when they are crouded together in too circumscribed a space.

When fires are kept, the chimnies have this double effect; but as every part of a ward cannot be heated by the means of fire places, so as to satisfy the sick, and as the local circumstances do not always admit of them, stoves have been occasionally made to supply their place. What has been thus gained on the score of the saving of fuel, and of the distribution of heat, has been lost on the score of the renewal of the air. When the construction of stoves is considered, it must be manifest that they cannot determine so voluminous a current of air, as that which is established by the means of a chimney.

In reality, the aperture by which the air finds its way into stoves, having a diameter of a few inches only, can simply attract a column of air of that dimension; insomuch that a greater quantity cannot be renewed in the wards, at the same time that the air, which is not within the boundaries of this current, is driven back on the beds and on the walls. As, in the rivers which flow with the greatest impetuosity, the water in the middle of their bed glides rapidly on, while that at the sides remains in a manner motionless; so likewise the air, thrown into action by any cause whatever, escapes through the outlets which present themselves in its passage, and repels, in the lateral parts of the wards, the layers in its vicinity. The latter, being effectually made to ebb, are renewed with difficulty, and preserve for a long time their noxious quality. It has accordingly been remarked, that the patients placed in these parts of the wards are exposed to more serious relapses, lapes, and cured with greater difficulty than the others. It therefore becomes necessary, to put in action, at the different points of the wards, an agent possessing a sufficient activity to embrace and draw off the totality of the volume of air contained in them.

A method which has been recently proposed to the council of health, and which has met with its fullest approbation, consists in applying to the funnels of the stoves at this time employed in the hospitals, the *aspirators*, or suckers, invented by Salmon, surgeon to the military hospital of Nancy. These aspirators are cones of canvas fourteen inches in length, forming a kind of trumpet, the large aperture of which has a diameter of ten inches, and which is terminated by another aperture of three quarters of an inch. This latter extremity is introduced into the funnel of the stove about an inch and a half, and is there solidly fixed. In proportion as the heat within the stove is augmented, the extremities of the aspirators which are within the funnel receive an additional warmth, and attract in the same degree the atmospherical air of the ward, which is constantly disposed to place itself in equilibrium with the warmer current of air circulating within the funnel. This attraction is made with great celerity, and in proportion to the mass of air that has acquired a mephitic quality.

This ingenious expedient has been attended by the most complete success, and is susceptible of further improvement. It renews the air which has not contributed to combustion, and renders the stoves, by which that element has been hitherto vitiated, capable of maintaining its salubrity. In the plate annexed to this article, A. is the stove; B. the funnel; and C C. the canvas *aspirators*, or suckers. The necessary explanations for the adoption and application of this contrivance have been given above. To facilitate its effect, vessels filled with pure water ought to be placed on the stoves, more especially on those heated with coals. It is unnecessary to add that it would be of the highest utility in manufactories, and in all public establishments in which stoves are employed.

Whatever may be the efficacy of the mode which has been thus pointed out, it cannot, however, be employed for the renewal of the air, unless during winter. It therefore becomes necessary to substitue something in its stead during the other seasons of the year.

Fire also affords us this help, by the application of the ventilating furnace which has been for several centuries employed in the coal mines. Instead of placing it, however, on the top of the building, a less dangerous and more commodious situation may perhads be found for it.

When the atmosphere is perfectly calm, the current of air is too weak to facilitate the escape of the portion withinside. In such a case, Maret, a physician of Dijon, has proposed to suspend, in the middle of the window the most favourably situated, a chafing dish filled with lighted charcoal, which, by rarefying the air at that point, may determine thither a current possessing a sufficient rapidity to traverse the ward, and carry off with it a part of the infected air.

It would be highly improper to neglect to open, every morning, and invariably on the side opposite to that from which the wind blows, the doors and windows of the wards. These outlets ought to be multiplied as much as possible; and corresponding ones ought to be made, to give a free access to the circulation of the air, more particularly while the beds are making, and the wards swept.

The renewal of the air may likewise be effected by forming, in the inferior part of corresponding windows, shutters contrived to open on a swing, so as to compress the air, which, having thus acquired a greater force, will give motion to the portion it will renew, and, by displacing it, will prevent the sick from being exposed in too immediate a way to the impression of cold.

The supplementary means which are best calculated to renew the air, and to diminish the causes of insalubrity, cannot be too much multiplied. A very simple one, the good effects of which have been demonstrated by the daily experience of those who work in the mine quarries, consists in boring, in the walls, and more particularly in the angles of the wards, corresponding holes from the floor to the cieling. By opening successively one beneath, and another above, opposite to it, taking care at the same time that the others should be closed, a current which sweeps away the stagnant air is established.

Experience demonstrates that the most turbid water becomes drinkable, and the most unwholesome air is fitted for respiration, by the means of a motion impressed on them. In either of these cases, it is constantly the air that has contracted the bad qualities, which are expelled and replaced by a purer air. Now, this advantage can only be obtained by the agitation of these two fluids, which are so essential to life. It would therefore manifest a criminal indifference indifference to neglect any thing which can give mobility to the air, and occasion its renewal.

It will be likewise expedient to establish vent-holes in the different parts of the wards, and to multiply them in proportion to their capacity, employing, for instance, an inverted trough provided with a tube rising from the floor to the ceiling, and a valve made to open and shut at pleasure by the means of a cord and pulley. It cannot be too often repeated, that dwellings in which the air is stagnant are as prejudicial to the health as marshy grounds.

During the summer, when the atmosphere is surcharged with heat, each of the wards may be provided with a large fan, which, being put in motion by the help of a cord, may agitate the air, force it to flow out in proportion as it is vitiated, and convey to the sick a salutary and refreshing coolness.

The use of thermometers ought to be adopted in hospitals; and it should be so contrived as that the temperature of the wards should never exceed from 66 to 68 degrees of Fahrenheit.

During the violent heats the passages leading to the wards should be frequently sprinkled with water; and, withinside, branches of trees recently cut should be distributed, with a view to the refreshing coolness which is so desirable and necessary.

As far as it may be practicable, trees, shrubs, and *inodorous* plants, ought to be kept in full vegetation in the vicinity of hospitals.

### *Chemical Means.*

It is not enough to have prevented the air within from contracting, by its stagnation and a want of communication with the external atmosphere, a noxious disposition; but it is likewise necessary to attack those morbific particles from which fatal effects result, even in the medium that has been just particularized.

It is known that there exist diseases, which, during the whole of their duration, are productive of emanations the more dreadful in their results, in proportion as the constitution of the air which receives them is vitiated; as the ceilings of the wards are low pitched; and as there are a greater number of individuals collected together. These emanations, these *germes*, which are even living after the destruction of the cause that gave them birth, fix and attach themselves to the walls, floor, sheets, blankets, articles of wearing apparel, and bedsteads. They have the dangerous faculty, of preserving for a long time their *deleterious* quality, and likewise of continually poisoning the air. Under these cicumstances, the means that have been pointed out above become insufficient to produce the *disinfection*; and it is therefore necessary to combine with them still more powerful agents.

Perfumes, of whatever kind they may be, are far from possessing the wonderful properties that have been ascribed to them, and afford but a treacherous security. During their ignition within a circumscribed space, they consume the portion of vital or pure air which they abstract from the atmospherical mass. When they are gradually consumed, so as to become carbonated, the more or less aromatic vapour they exhale is soon confounded with the air it tends to vitiate. When it is inspired in a mass by those of the patients who receive its earliest impressions, it may occasion derangements of the animal economy. This vapour does not supply a new air; and, being extraneous to that with which it blends itself, does nothing more than disguise the bad smells, without destroying them. No time ought therefore to be lost in banishing perfumes from the apartments of the sick.

This opinion relative to aromatic fumigations is not in contradiction with that of the ancients. The forests they consumed with a view to purify the air of infected countries; and the large piles, composed of odoriferous woods, the flame of which was directed towards the cities where a contagion prevailed, were nothing more than immense fires purposely employed to give to the air a greater mobility, and to restore to it, by its renewal, the purity and elasticity of which it had been deprived by any cause whatever. This was invariably done on the supposition that the air was the vehicle of all pestilential scourges.

In several hospitals vinegar has been employed in preference to aromatic substances. It has been the practice to throw it on a heated fire shovel, for the purpose of expelling the infected smells, and neutralizing the putrid miasmata dispersed in the atmosphere. It is, however, erroneous to imagine, that, when it is thus decomposed and reduced into vapour, it possesses any such property. In the same way as the perfumes, it does nothing more than surcharge the air, while it diminishes its elasticity, and renders the infected odour, which it was intended to correct, still more sensible.

It is not but that vinegar, when thrown into expansion in a bottle with a large orifice, may, in common with all the the acids in a gaseous state, be capable of forming combinations with the putrid ammoniacal miasmata, and, by their destruction, may restore to the air, in which they were in a manner dissolved, its purity and elasticity. But in these cases, its efficacy, relatively to which there is but one opinion, cannot be compared with that of the acetic acid, or radical vinegar; and the latter is still inferior to the agent we now proceed to notice.

Among the means which chemistry has suggested, and which have been employed, to bring about this depuration, with a success which, at a first glance of the subject, seems to surpass all belief, we shall cite the process employed by Guyton Morveau in 1773, in the Cathedral of Dijon, infected to such a degree by the bodies which had been dug up, that it was abandoned by the inhabitants.

This process consists in diffusing in the atmosphere the muriatic acid (acid of sea salt) in a gaseous state, disengaged by the intervention of the sulphuric acid (oil of vitriol). The following is the method of freeing from infection a ward containing from forty to fifty beds.

After having removed the sick into another ward, place in the middle of the empty ward, the windows and doors of which ought to be closed, a furnace provided with a small iron pot half filled with sifted wood ashes, on which is to be laid a glass or earthen vessel containing nine ounces of muriate of soda (common salt) slightly moistened, with half an ounce, at the most, of plain water.

The fire having been lighted, and the vessel heated to a proper degree, four ounces of sulphuric acid, or the oil of vitriol of the shops, are to be poured on the sea salt. The sulphuric acid will act instantaneously on the latter, the acid of which will be made to expand. The operator, who ought to be the apothecary in chief, or one of his assistants well acquainted with the mode of performing chemical operations, should now retire, shutting the door, and taking possession of the key.

He should not enter the ward until twelve hours after, when the doors and windows ought to be opened, to establish currents of air, and to get rid of the portion which may be still charged with the acid.

This process will be rendered of still greater utility, by the application of it to the wards still filled with the sick, as often as the medical men may deem it expedient. Thus, when it has been ascertained that the air of a ward is charged with animal miasmata, and is in want of this excellent purifyer, it will suffice to employ a third of the mixture.

mixture above pointed out, or even less. In this case the operator, having the chafing dish in his hand, should proceed, with more or less promptitude, to every part of the ward, beginning as soon as the gas is thrown into an expanded state. As soon as the ward is deemed to be sufficiently filled with the muriatic acid gas, the apparatus should be carried to the privy, to the end that the latest gaseous portions which the mixture may continue to supply, may serve to neutralize the putrid ammoniacal gases which are constantly developed in places of that description.

This operation, which is not attended by any disagreeable sensation, is nevertheless sufficient to cleanse and purify a ward. It may be applied daily, and even more frequently, in a partial manner, to wards in which one or several patients, labouring under gangrenous affections or other putrid diseases, may throw off dangerous miasmata. In an urgent case, provided the concentrated muriatic acid (fuming marine acid) should be found in the dispensary, the same effect may be obtained by carrying an open bottle filled with this fluid in the wards. If it should not be sufficiently concentrated, it may be warmed with a view to bring it into a gaseous state. Finally, these processes may be repeated as often as it may be deemed necessary by the professional men, as was formerly the practice in the case of the useless and even dangerous aromatic fumigations.

Before the operation is entered on, it is expedient that the precise state and condition of each of the patients should be correctly ascertained, to the end that those who have the care of them may, when the atmosphere of the ward is charged with the muriatic gas, be enabled to observe with greater certainty, whether the individuals exposed to its action are subjected to any change, which may, directly or indirectly, be ascribed to this destroyer of putrid miasmata. This precaution will tend to augment the confidence of all, and, perhaps, to perfectionate the application of the means.

The surgeons should be careful not to leave their instruments in the ward in which the muriatic acid is thrown into expansion, seeing that it attacks steel, which it rusts in an instant. On another hand, the apothecaries, to the end that nothing may be wasted, ought to collect the remains of the different mixtures, from which they will obtain, as a product, the sulphate of soda.

It may readily be conceived, that when it is found necessary to diffuse a large quantity of the muriatic gas, its disengagement ought not to be attempted in wards which, together together with the effects they contain, are to be freed from infection, until after the patients have been removed. The only mode of effecting this, is to have constantly, in each hospital, a *change ward* for the reception of the patients belonging to the ward it may be deemed necessary to free from infection. This ward ought not, on any pretext whatever, to be applied to any other than the above salutary purpose; and in the principal hospitals two wards ought to be kept for this operation.

The ward which has been thus purified, will in its turn, answer the purpose of a *change ward*, and thus successively, until all the wards shall have been freed from infection, and the miasmata disseminated on the surface, and in the atmosphere of the hospital, shall have been neutralized and destroyed: — until, in short, the air shall have been entirely renewed.

In the ward in which the operation is performed on a large scale, the blankets, mattresses, clothing, and, in general, all the pieces of linen or woollen cloth which may have been employed in particular diseases, ought to be exposed to the action of the muriatic gas, in such a way as that the vapour may reach each of the surfaces of the substances in which putrid miasmata may lurk. The same thing ought to be done in the passages and avenues leading to the wards.

The oxygenated muriatic acid possessing, as Fourcroy has observed, a still greater energy, ought to be preferred in this process. Accordingly, when the oxyd of manganese can be readily procured, a small quantity of it may be added to the above mixture. For this purpose, the metallic oxyd in question ought to constitute a part of the supplies of the dispensaries.

The combustion of sulphur has been employed, not unsuccessfully, with the same view. But the sulphurous gas which is disengaged is not managed with the same facility, and does not, besides, rise so readily into the upper regions of the air.* It cannot therefore be substituted, without detriment, to the vapours of the muriatic acid, which, by their extreme expansibility, readily diffuse themselves in the upper and lower layers of the atmosphere, seizing with avidity on the putrid miasmata which are there condensed, and which seem to be of the nature of ammonia (volatile

* This observation, which is extremely important, applies equally to the *nitrous* acid.

(volatile alkali). These miasmata the muriatic acid attacks, wherever it encounters them. It is expedient, however, not to neglect the combustion of sulphur.

The means of explosion and deflagration hitherto employed, to destroy contagion, such as inflamed nitre, gunpowder, and the discharge of fire-arms, act merely by their mechanical effect, and do nothing more than displace and change the air for the moment. Their efficacy is but of a short duration; and independently of this consideration, the only gases they disengage are the azotic and the carbonic acid. Lime-water itself, by which the latter of these gases is absorbed, does not appear to annihilate the morbific miasmata.

The complicated ventilators, on which so many praises have been bestowed, if duly appreciated, will be found to possess but a comparatively trivial advantage. By the space which they occupy in the wards, they are rather an incumbrance, and an obstacle to the free circulation of the air, than an assured expedient for its complete renewal.

At this time, when chemical knowledge has been successfully applied to our prime necessities; when it is as easy to analyze the air as other fluids; when its nature can be ascertained in an instant; and when the specific qualities which it needs, to contribute to the maintenance of life, can be restored to it; the medical men belonging to hospitals ought to consider it as one of their most essential duties, to ascertain, from time to time, the temperature of the air of the wards, taken in the angles, and at the head of the beds.

The experiment is a very simple one. It consists in entering the ward, provided with two phials, one of them filled up to its mouth with pure water, and the other with lime water. The former is to be emptied at the spot where the quality of the air is to be ascertained; and the one half of the lime water contained in the second bottle to be added instantly after. The vessel having been nicely closed and agitated, the quantity of the precipitate, and the promptitude with which it is thrown down, will serve to determine the necessity and urgency of the employment of the muriatic gas; seeing that the knowledge recently acquired, relatively to the nature of the gases, renders it highly probable, that in the wards where a suspicion of insalubrity is entertained, the putrid miasmata are constantly accompanied by a considerable quantity of the carbonic acid.

Lime water presenting the most economical and efficacious mode of freeing the air from the carbonic acid gas which is necessarily produced by a great number of persons collected together; and this acid being so much the more dangerous as it is retained by its gravity in the inferior region of the air; vessels filled with lime water ought to kept in each of the wards. The promptitude with which the pellicle is formed, is the best eudiometer to ascertain the presence of the carbonic acid gas; since those of Fontana, Volta, and Scheele, merely point out the air from which vital air has been drawn.

*Conclusion.*

It results from what has been laid down, that as cleanliness has a decided influence on the salubrity of hospitals, its strict observance in every particular will render the employment of the mechanical and chemical means which have been pointed out, either less frequently necessary, or more efficacious in their results. Accordingly, to renew the air of the wards, and to destroy the mephitic vapours which usually abound in them, it ought to be observed:

1st. That the hospitals should be freed from every source of infection; that the sick should not be crouded together in too great numbers; that the utensils destined for their different uses should be perfectly cleansed; that the hospital great coats and blankets should, together with the various articles of clothing, be subjected to the action of the muriatic acid, or sulphurous gas, whenever they have been worn or employed by patients labouring under contagious diseases; that the body linen, sheets, napkins, &c. should be well washed; and the walls and floors swept daily.

2dly, That a well directed fire being the most certain mode of preventing the stagnation of the air, of establishing strong currents, of renewing it, and augmenting its motion, it is necessary to multiply these currents, in proportion to the extent and form of the building, and constantly to give them such a direction as will enable them to expel the foul air at each of the points of the wards; that the *aspirators*, or suckers, fixed to the funnels of stoves, constitute at this time the instruments best calculated to produce such an effect; that every opportunity ought to be embraced to open the windows, as well as the apertures made in the doors, aud at the angles of the walls; and that the vegetation which nature employs to maintain and re-establish the salubrity of the air, ought to be comprehended among the means employed to maintain the salubrity of hospitals.

3dly, and lastly. That the means of cleanliness, and the mechanical mechanical means, inteuded to produce the effects that have been pointed out, are sometimes not sufficiently powerful to destroy the putrid ammonical miasmata which particular diseases disseminate in the atmosphere; that the common muriatic gas, and the oxygenated muriatic gas, eminently possess the property of seizing these miasmata wherever they may have fixed themselves, and of decomposing and neutralizing them; that this process ought to be performed on a large scale, and successively in all the wards of the hospital, by the means of an empty ward to be denominated the *change ward,* which ought to be constantly and exclusively reserved for the reception of the patients belonging to the ward intended to be purified, and by the aid of which the totality of the hospital may be sweetened, and freed from so deleterious a principle. As lime possesses, however, the property of readily absorbing the carbonic acid gas, vessels filled with lime water, which should be agitated from time to time, and renewed as the circumstances may require, ought to be placed in the extremities of the wards.

The Council of Health being desirous to avoid pointing out to its fellow labourers a process with which many of them may be unacquainted, without having at the same time ascertained its efficacy, by trials made in the establishments within its reach, sent several of its members to the hospitals of St. Cyr, St. Dennis, and Gros Caillou, to institute the necessary experiments. The result of these experiments has incontestibly proved, that the mode proposed to free from infection the wards of hospitals by the muriatic acid gas, may be executed without inconvenience, and with the utmost advantage, as well in inhabited as in empty wards, observing, however, to disengage in the former, a smaller quantity of gas.

The expedients which have been proposed are not, as has been already observed, confined to hospitals; but may be rendered highly useful in barracks, ships of war, prisons, houses of industry, and, in general, in all the asylums where a considerable number of persons are assembled. These establishments, the greater part of the inmates residing in which labour under either physical or moral derangements, may be equally infected by a vitiated air, and may require the employment of the same precautions, to extinguish this source of exhalations that are always pernicious.

Before we conclude, we deem it essential to remark, that in presenting a great number of means to prevent the infection fection of the air in hospitals, and to free them, whether from mephitic vapours, or from putrid miasmata, it has been our aim to render them supplementary to each other. Unquestionably, they do not all of them possess the same energy; but the effects of each are analogous; and too many sure weapons cannot be resorted to against such an enemy. Their employment will lead to a due appreciation of their respective merits, and of the greater or smaller share of attention to which each of them may be entitled, according to the local circumstances. Such is our reply to those who may consider any part of these instructions to be superfluous.

To the powerful considerations of humanity, and of what we owe to our suffering fellow creatures, the Faculty, ought to unite what their own interest demands. Living in a manner in the very focus of morbific emanations, they incur the risk of becoming daily, by a neglect of the precautions that have been prescribed, the victims of the scourge, the preservative and the remedy for which are the objects of these instructions.

Signed. DAIGNAN, BAYEN, PARMENTIER, HEGO, HEURTELOUP, LASSIS, PELLETIER, THERY, CHEVALIER, DUBOIS.

BIRON, *Secretary.*

## Figures and Tables

**Figure f1:**